# Wessex Branch of the Association of Clinical Pathologists Winter Meeting 1992

**Published:** 1992-12

**Authors:** 


### Wessex Branch of the Association of Clinical Pathologists

**The winter meeting of the Wessex Branch of the Association of Clinical Pathologists was held on 10 December 1992 at the Postgraduate Centre, Southmead Hospital. After the business meeting (President: J D Davies; Secretary: E F Mackenzie; Treasurer: N B N Ibrahim), and a scheduled address by a member of the Executive Committee of the Royal College of Pathologists, the following oral papers were presented by trainee pathologists from the South West Region.**

### QUANTIFYING THE OCCULT

M B Salzmann, A G Lim*, K A Muhiddin*,
T K Daneshmend*
Department of Chemical Pathology
*Department of Medicine
Royal Devon and Exeter Hospital, Wonford.

An assay based on the fluorescent properties of di-carboxylic porphyrins derived from the haem moiety of haemoglobin was used to quantify blood loss in faeces.

Patients receiving thrombolytic therapy - streptokinase or thromboplasminogen activator - following myocardial infarction were studied. Haemoglobin levels in random faecal samples were compared with controls, who did not receive thrombolysis, admitted to the CCU during the same time period.

Mean peak measured faecal haemoglobin in the treatment group (n = 36) was 5.09 mg/g stool (SD = 7.50, SE = 1.25) and in the control group (n = 19) was 1.45 mg/g (SD = 1.30, SE = 0.30). The difference was statistically significant ($p < 0.05$). 25/36 (69%) in the treatment group and 2/19 (10.5%) in the control group had levels greater than 2 mg/g, the quoted upper limit of normal, in at least one sample. It is concluded that thrombolytic therapy is associated with increased GI blood loss although in none of the patients studied was the magnitude sufficient to cause haemodynamic compromise.

There are circumstances under which quantitative determination of faecal blood loss should be considered advantageous because of the limited sensitivity and specificity of the traditional, qualitative, guaiac impregnated slide tests. Porphyrin fluorescence analysis is a practical alternative to radioisotopic labelling of red blood cells to achieve this.

### ITS A MELANOMA - WHAT MORE DO YOU WANT!

H S Rigby
Department of Histopathology
Bristol Royal Infirmary

The potential for metastasis of melanoma can be assessed using clinical and histological prognostic variables and several prognostic models have been developed.The precision of such microstaging depends on the number and power of variables although a few of these such as thickness are sufficiently powerful to be considered as single variables in clinical use.

One hundred histological reports of excision specimens of stage 1 invasive melanomas from files were analysed to see how often basic histological features were recorded. None of the one hundred reports contained all the relevant histological data. In 15% of the reports there was no comment on the degree of excision. In many of the reports microstage attributes that have been shown to be prognostically important much as mitotic count or ulceration were omitted. A standard method of reporting invasive melanomas is advocated which includes these attributes and will allow some degree of survival probability to be made.

### THE DIAGNOSIS OF MYOCARDIAL INFARCTION

Christine A Neil
Department of Clinical Chemistry
Southmead Hospital

This study compared the sensitivity and specificity of various markers used to make the diagnosis of myocardial infarction. 50 patients admitted to a coronary care unit with a history of chest pain had frequent blood samples and ECG's taken during the first 24 hours. The ECGs were read blind by a cardiologist and the venous samples, after being stored at -70C, were analysed for Creatine Kinase, CK-MB, Myoglobin and Troponin-T. A full clinical history was obtained and a record was kept of all tests initiated by the admitting physicians and the diagnosis they reached on the basis of those tests. Data are presented outlining the ECG/biochemical criteria used to make the diagnosis, the misdiagnosis rate and the mortality rate in the different groups. The sensitivity and specificity of the individual markers were reviewed. A control group of 25 patients without chest pain had 6 hourly blood samples and a minimum of 1 ECG during the first 24 hours.

Data are presented on the ability of various markers to detect minor myocardial damage and their possible use as prognostic indicators.

### INTERLEUKIN-4 PROLONGS SURVIVAL IN CULTURE OF B CELLS PROM B-CLL PATIENTS; THE ROLE OF APOPTOSIS

A Mainou-Fowler, J A Copplestone and A G Prentice
Department of Haematology
Derriford Hospital, Plymouth

The survival in vitro of peripheral blood (PB) B cells from B-CLL patients was markedly enhanced in the presence of interleukin-4 (IL-4) as measured by apoptotic rate. The mean percentage (range) reduction of cells showing morphological apoptotic changes using acridine orange was 52% (28-77) on the first day, 40% (35-48) on the third and 30% (16-52) on the sixth day of culture with IL-4 and activation (PBW). Similar results were obtained without PWM. No reduction in apoptosis with IL-4 was seen in normal PB B cells. In eight further experiments, apoptosis was measured by incorporation of propidium iodide (PI) into isolated nuclei using FACSCAN analysis. The % (range) reduction with IL-4 and PWM was 60% (45-76) on the second day, 40% (12-58) on the fourth day, 36% (19-52) on the sixth day of culture. Agarose gel DNA analysis confirmed apoptotic ladder patterns with band intensity in proportion to the % apoptosis as determined by the other two methods. In conclusion, in vitro, IL-4 has anti-apoptotic effect on PB B-CLL cells. This effect was not associated with earlier proliferation in response to IL-4 or PWM.

### THE USE OF PCR IN LYMPHORETICULAR DISEASE

M Ilyas
Department of Histopathology
Bristol Royal Infirmary

The development of techniques such as Southern blotting, in situ hybridisation, northern blotting and the polymerise chain reaction (PCR) have led to the identification and understanding of consistent molecular "events" within certain disease processes. These techniques and events can be used as diagnostic aids.

The histological differentiation between benign and malignant lymph node biopsies and extra-nodal lymphoid infiltrates is notoriously difficult. One of the best ways of identifying malignancy is to show monoclonality. During differentiation of lymphocytes one "event" is rearrangement of the T cell receptor (TCR) gene and the Immunoglobulin (Ig) gene. Each rearrangement is cell specific

This presentation shows development of PCR to aid diagnosis in lymphoid malignancy. DNA has been extracted from as little as 3x5 micron thick sections of formalin fixed tissue. The suitability of the DNA for amplification has been tested by amplifying the HGPRT gene. To show monoclonality of a given lymphocyte population the CDR III region of the Ig heavy chain gene has been amplified. This region undergoes both nucleotide insertion and point mutation during rearrangement. Thus each lymphocyte has a unique CDR III size and sequence. The presence of a single sized band on electrophoresis after PCR is evidence of monoclonality. The success with fixed tissue means that archival material is now available for analysis and research.

### MUCOCOELE-LIKE TUMOURS IN THE BREAST: A WARNING TALE

J Kulka and J D Davies
Regional Breast Pathology & QA Unit, Bristol

Mucocoele-like tumours of the breast have only recently been recognised as a morphological entity. They consist of expanded duct-like spaces filled with mucin, which often extravasates into the adjacent connective tissue. Initially they were regarded in the United States as a benign and incidental finding. Later reports have demonstrated association with atypical ductal hyperplasia and mucoid carcinoma.

We report two cases. One was found as an impalpable mammographic lesion in the national Breast Screening Programme, and the other was detected in a retrospective survey of cases indexed as "benign fibrocystic disease". Shortly afterwards the second case, diagnosed 10 years ago, was found to have an invasive carcinoma of non-mucinous type. Neither impalpability nor association with a non-mucinous carcinoma have been recorded before. We propose consideration of a contrary interpretation of mucocoele-like tumours: it would seem prudent to regard them as ductal carcinomas in situ of mucin-secreting type. It may well be that these lesions are underdiagnosed. It is for this reason that attention is called to them in this communication with the hope that a better understanding of their true status may be achieved.

### APPROACHES TO DEALING WITH INCREASED OUT OF HOURS WORKLOAD

A Gunneburg
Department of Clinical Chemistry
Southmead Hospital

A pattern of increasing total out-of-hours requests received by a Clinical Chemistry laboratory over a five year period was identified. Occasional audit revealed that in about 30% the reason for urgency was unclear. Altered clinical working practises resulted in some samples arriving later in the day and becoming "urgent" because results were required pre-operatively on the following day. Analysis of the figures in the fortnight periods preceding and following the change-over of house officer staff showed an increase of about 20% in the out-of-hours workload following the August new house intakes while no consistent increase could be detected following the February change overs.

The overall increase in the out of hours workload was met by a more flexible redeployment of staff with the establishment of an extended working day. This mirrored the altered requesting pattern by the clinicians and significantly reduced the number of out of hours requests. The August 1992 intake of house officers were issued with guide-lines on the use of the Clinical Chemistry out of hours service and an invitation to visit the laboratory. During the first week of August all out of hours calls on four nights including the first weekend were answered by a chemical pathologist who took details of the requests and, where appropriate, discussed the reasons for their urgency with the clinicians. These measures appeared to limit the workload increase to 6%.

### ACCUMMULATION OF WEAR DEBRIS FROM JOINT PROSTHESES IN LYMPH NODES LIVER AND SPLEEN - A CAUSE FOR CONCERN?

C P Case, V G Langkamer, P Heap, M Palmer, C Collins, L Soloman
Department of Histopathology
Southmead Hospital
Departments of Histopathology and Orthopaedic Surgery
Bristol Royal Infirmary
Department of Anatomy and Geology
Bristol University

Metal wear debris formation is a well known complication of joint replacement. There is concern that these metals, which are known in other situations to have both toxic and carcinogenic properties, might be responsible for local tumours and the increased risk of lymphoma and leukaemia that have been described in patients with hip replacements. In this study post mortem tissues from five patients with joint replacements and six control patients were studied with light and electronmicroscopy and mass spectometry. Our results show a greatly elevated level of metals, including Nickel, Chromium, Cobolt and Iron, in the lymph nodes, liver and spleen of patients with joint replacements.No accumulation was found in other organs including lung, kidney and brain. These metals were derived from the surface of the implant and were stored within macrophages.

We conclude that there is a specific accumulation of metals at those sites shown to be at risk of carcinogenisis in the literature. Whilst there is no direct proof that metal in the form of debris is toxic or carcinogenic there must be concern about its accumulation in the reticulo-endothelial system, especially in younger arthoplasty patients.

### LACTOFERRICIN

E M Jones
Department of Medical Microbiology
Frenchay Hospital

An antimicrobial peptide called Lactoferricin has recently been described by a research group from Japan. Tomita et al produced the peptide by acid-pepsin hydrolysis of bovine lactoferrin, and iron-binding protein also found in tears, gastric juice, genital and bronchial secretions. Tomita et al demonstrated that Lactoferricin has antimicrobial activity and subsequently they went on to sequence the peptide. The purpose of this study was to confirm Tomita's findings and expand on his work including clinical investigations. Lactoferricin was kindly supplied by Tomita of the Morinaga Milk Industry - Japan. The purity of the supplied Lactoferricin was confirmed by HPLC and internal sequencing, this work was done in collaboration with Dr. Bloomberg at the Biochemistry Department of the University of Bristol. A wide range of organisms were tested and activity was demonstrated against both gram negative and gram positive organisms. Yeasts were also sensitive to Lactoferricin.

The inoculum and ionic strength were found to be important variables; Lactoferricin's activity was markedly decreased when inoculae or ionic strengths were used. The results obtained so far confirm all the findings of Tomita et al. In addition we have produced data for more organisms demonstrating a wide spectrum of activity but also showing that not all organisms are susceptible and that there is a significant amount of strain variation within species. The clinical significance of these findings may be wide ranging. Of particular interest is the possibility that Lactoferricin is actually produced in the infants stomach following a milk feed resulting in a degree of antimicrobial activity in the small intestine. It is worth noting that human milk contains a higher concentration of lactoferrin/100g dry weight than cow's milk. Could Lactoferricin be the reason why breast is best? Lactoferrin is also found in many other bodily secretions, it may therefore be possible to find Lactoferricin there also.

### CATIONIC IMPREGNATION BY HEAVY METAL SALTS OF BREAST TISSUE ENHANCES SOFT-TISSUE MAMMOGRAPHIC IMAGES

E K Dankwa, J D Davies*
Cambridge Military Hospital, Aldershot, and
*The Regional Breast Pathology Unit, Bristol BS10 5NB

Preliminary radiography of slices from breast biopsies draws attention to relevant pathological changes underlying mammographic abnormalities. Nearly a third of the lesions are due to fibro-parenchymal changes (mass lesions or distortions of architecture). They cause many difficulties in appropriate grey-scaling if quantitative morphometric analysis is contemplated. In pilot studies we have found that post fixational impregnation of breast tissue by 2% w/v metallic salt solutions appears to improve radiological contrast of the fibro-parenchymal tissue in slice mammograms of specimens.

We now report a detailed and replicated (n = 14 to 16) study using eleven metal containing inorganic salts undertaken before and after possible radiological enhancement using radiographs of reduction mammoplasty tissue (kindly supplied by the Department of Histopathology at Frenchay Hospital). the changes in radiographic density (assessed in a variety of modes by CUE2 densitometry software, Messrs. Olympus) of the fibro-parenchymal tissue showed the following approximate ranked order of radiodensity enhancement for fibro-parenchymal tissue by salts containing: Hg, Pb, Ur, Zn, La, Co, Cr, Mn, Mg, Ca, Bo. No discernible enhancement was found for the last four elements. Use of Os tetroxide led to disintegration of the tissue. Histology and histochemistry showed that all the metal cations, except uranyl acetate, were deposited in the mammary fibro-parenchyma, but not in adipose tissue. The results may reflect the strength of metallic complexing between proteins and dyes, and, the radiodensity of the metals.

Although the mode of chemical bonding between components of non-adipose tissue and the metal cations is unknown, the method is potentially useful in its radiological enhancement of fibro-parenchymal structures. It has application for quantitative morphometric evaluation of specimen mammograms. Use of the method with three-dimensional imaging techniques may allow computer assisted rotation of the lesions, thus enabling more detailed comparison between radiological and pathological findings.

### WHO WAS SAGO?

B F Warren, H S Rigby
Department of Pathology, University of Bristol

Teaching of medical students is aided by all manner of computers and electronic gadgetry. One part of student teaching which has not changed in Pathology is the use of food terms in descriptive macroscopic pathology. These food terms were established many years ago. They now bear little relationship to the modern medical students diet to the extent that most medical students are unaware of the appearance of these foods, or indeed, in the case of sago, are unaware that it may be eaten at all! We presented 30 medical students with a questionnaire which was designed to record an individual student's knowledge of the relationship between a classical appearance of disease and a food substance. 92% of students had seen the diseased organ which fitted the description, but had not seen the related food item. Indeed many students did not know what the related food item was! This study highlights the need for change in this small but important aspect of the teaching of descriptive pathology. Modernisation of food terms to comply with the world of modern diet should be carried out in the pathological text books forthwith.

### A PRIMATE MODEL OF THE COLITITS-CARCINOMA SEQUENCE

P E Watkins, G R Pearson, & B R Warren
School of Medical Sciences, University of Bristol

The study of the colitis-carcinoma sequence has been hampered by the lack of a suitable model. The cottontop tamarin (a New world primate) is unique in developing a spontaneous colitis which bears a close resemblance to human ulcerative colitis as assessed clinically, endoscopically, histologically and in its response to treatment. It also develops liver disease closely resembling sclerosing chlangitis.

23 cases of colonic carcinoma complicating established ulcerative colitis in the tamarin are reported. All were flat and mucinous. The majority were right-sided. All showed early local spread with involvement of the pancreaticoduodenal lymph node. In 3 cases the tumours were microscopic but had produced lymph node metastases. The only differences between these animal tumours and those complicating human ulcerative colitis are: 1) lack of hepatic metastases, and 2) relative rarity (2 out of 23 cases) of preceding dysplasia. This communication describes the first reliable model of carcinoma complicating ulcerative colitis. It is envisaged that it will permit controlled studies of premalignant changes in inflamed colonic mucosa.

### AN UNUSUAL CASE OF INTERFERENCE IN THE IMMUNO DIAGNOSTIC SYSTEMS (IDS) COATED TUBE THS ASSAY

P H Thomas
Department of Chemical Pathology
Bristol Royal Infirmary

CW presented failure to thrive. Thyroid function tests in January 1992 were reported as TSH 18.8 mu/l and free T4 11.6 pmol/l. At follow up TSH was >50 mU/l and Free T4 25.4 pmol/l. When the same sample was assayed 7 days later the TSH was 18.3 mU/l and Free T4 25.5 pmol/l, after a further 4 days the THS had fallen to 8.3 mU/l. The analytical validity of these results was investigated.

The Free T4 result was confirmed on two subsequent samples and by comparison with the DELFIA total T4 assay. Repeat samples showed a similar decrease in TSH. Studies showed no evidence of in-vitro degradation of TSH. Dilation of a fresh sample showed non linearity suggesting a analytical interference in the TSH assay. This IRMA assay uses a goat polyclonal antibody and a solid phase mouse monoclonal antibody. Addition of mouse/goat serum suggested the presence of a anti-goat heterophilic anti body. Assays which use a dual mouse monoclonal antibody showed no decrease in measured analyte with time. Although the interfering antibody appears to precipitate out with time the presence of precipitated immunoglobulin has not been shown.

**CD 30 IN CUTANEOUS LYMPHOID INFILTRATES**

E Burd, N Rooney, N Cope, C Kennedy
Departments of Histopathology and Dermatology,
Bristol Royal Infirmary and
The Department of Histopathology
Royal Devon and Exeter Hospital

CD 30 previously called the KiL antigen is an activation molecule expressed on Reed-Sternberg cells and the large cells in lymphomatoid papulosis and anaplastic large cell lymphoma. It is also found in the tumour stage of mycosis fungoides. In 1987 Ralfkaier et al demonstrated the presence of CD 30 in 3/14 cases of mycosis fungoides. To evaluate the specificity of CD30 staining we immunostained a range of paraffin processed inflammatory skin biopsies with a panel of antibodies (CD30), CD68 (histiocytes), (CD20 B cells), CD45R0 (T cells), S100 (Langerhans and dentritic cells). The cases were chosen from the BRI archives from 1981-1984 to allow 8 year follow up and included mycosis fungoides (15), Jessner's (10) eczema (10) lymphomatoid papulosis (2) large cell lymphoma (8). The diagnoses were reviewed and the positive cells in the infiltrate scored as, 1(<10%), 2(10-50%), 3(51-100%). The results were as follows. MF 0=6, 1=6, 2=3; eczema 0=9, 1=1; Jessners 0=10; LyP 0=1, 3=1; LCl 0=3, 2=2, 3=3. These results indicate that CD 30 is valuable in separating benign lymphoid infiltrates from tumours derived from the activated T cell i.e. lymphomatoid papulosis, mycosis fungoides, and anaplastic large cell lymphoma. CD30 should be incorporated in antibody panels for the analysis of cutaneous lymphoid infiltrates.

### Hospital Art

**David Levine**
September 1992

For a few days it was a glorious distraction from the aims and the targets, the muddles and the meetings. For a few days we were able to argue and discuss a matter of such importance that Management was moved to act fast to prevent serious harm coming to the local people.

The first inkling of a problem came at nine o clock on a Monday morning, The Redruth diabetic clinic has long been the breeding ground for martyrs; set in a medical no man's land, the watershed between West Cornwall and Truro, it is possible for the doctor there exiled to feel strangely protected from attack. The impossible task of modifying recalcitrant behaviour away from all support allows one to feel distanced from other problems, Telephone calls pleading medical disasters elsewhere in the district can be met with a spiritual calm normally requiring limbic doses of Glenmorangie.

I have to admit that a call so soon after the start of the clinic did stir a faint unease. Even in the seconds before hearing the voice it is possible to consider enough trouble to cause real concern; too early for GPs to have got a new problem but too late for a residual mess from the weekend. Curiously profound relief at hearing the voice of the Penzance nursing officer, army trained, straight to the point, frontal assault.

"Had I seen it?"

"No"

"There had been complaints from The Public, what was I going to do about it?"

"Nothing, I haven't seen it.,"

"Had I chosen it?"

"No!"....... but I wish I had.

"When was I coming back to Penzance?"

"Hmmm not until late evening,"....... I'm beginning to enjoy this.

A morning diabetic clinic followd by an afternoon gastroenterology clinic leaves numbness, the state in which best to admire the pointed breasts, the handless, footless and faceless naked bodies, the 8'x4'' canvas vibrant and challenging.

Over the last four years we have been lucky to be able to display the works of contemporary Newlyn School artists on the hospital corridor walls' I have been happy to let the gallery make the choice each year' Clearly it was felt that we were now able to appreciate nude female forms in joyful outdoor callisthenics.

It is easy to imagine the impact of 'Les Demoiselles d'Avignon' in 1906. Eighty six years later you may find it harder to understand the embarrassment and anger which was caused by our innocent painting.

The exchanges over the next two days became steadily less coherent and more acrimonious.

"The public don't want to see this sort of thing.

"But it was chosen by gallery staff who are members of the public".

"That's different; they're interested in art."

"Ah yes."

"You don't expect to see naked women in a hospital."

"Really?"

"Think how it could upset a patient who's had a mastectomy."

"The mastectomy nurse thinks it's very good."

"Shut up!"

Only three complainants were ever identified, all members of staff, but a manager was persuaded to have the picture removed, The subsequent gentle outrage from the considerable numbers who resented the censorship was some compensation.

A replacement picture by the same artist was not a threat to decency; I wonder though whether the hospital gardener wouldn't complain about those aggressive looking plants. Here he comes now....... Go on, make my day.

